# Perturbation of the preterm human immune system in early life

**DOI:** 10.1172/jci.insight.201342

**Published:** 2026-04-21

**Authors:** Benjamin A. Fensterheim, Michelle L. McKeague, Divij Mathew, Shwetank, Ajinkya Pattekar, Matthew Lee, Zahabia Rangwala, Sean Nasta, Macy C. Kee, Cynthia Clendenin, Zachary Martinez, Caroline Diorio, Allison R. Greenplate, Krithika Lingappan, E. John Wherry

**Affiliations:** 1 Department of Pediatrics, Division of Neonatology, Children’s Hospital of Philadelphia, Philadelphia, Pennsylvania, USA.; 2 Institute for Immunology and Immune Health and; 3 Department of Systems Pharmacology and Translational Therapeutics, University of Pennsylvania Perelman School of Medicine, Philadelphia, Pennsylvania, USA.; 4 Department of Pediatrics, Division of Oncology, Children’s Hospital of Philadelphia, Philadelphia, Pennsylvania, USA.

**Keywords:** Development, Immunology, Inflammation, Adaptive immunity, Cellular immune response, T cell development

## Abstract

Although inflammatory complications are common in preterm infants, the effects of these conditions on neonatal immune development remain poorly defined. We therefore investigated whether severe bronchopulmonary dysplasia (BPD) and systemic infection, 2 major complications of prematurity, produce distinct immune signatures and change immune composition over time. We performed longitudinal high-dimensional immune profiling of residual whole blood from 38 preterm infants sampled every 2 weeks, along with 10 term infants at birth. Preterm infants with severe BPD showed a progressive increase in Th17-polarized CD4^+^ T cells, neutrophils, and Th17-related cytokines compared with age-matched infants with moderate BPD. In contrast, some preterm infants with systemic bacterial or viral infections mounted exceptionally robust CD8^+^, CD4^+^, and γδ T cell responses, with oligoclonal expansion, terminal differentiation, and coordinated plasma cytokine shifts that persisted well beyond resolution of infection. These findings demonstrate that different preterm comorbidities imprint the neonatal immune system in divergent ways. Thus, comprehensive and longitudinal immune profiling may not only identify connections between clinical inflammatory complications and underlying immune pathways but also reveal potential targets for intervention.

## Abstract 2

In preterm infants, lung disease and infections drive distinct changes to the immune system that expose new potential targets for therapy.

## Graphical abstract

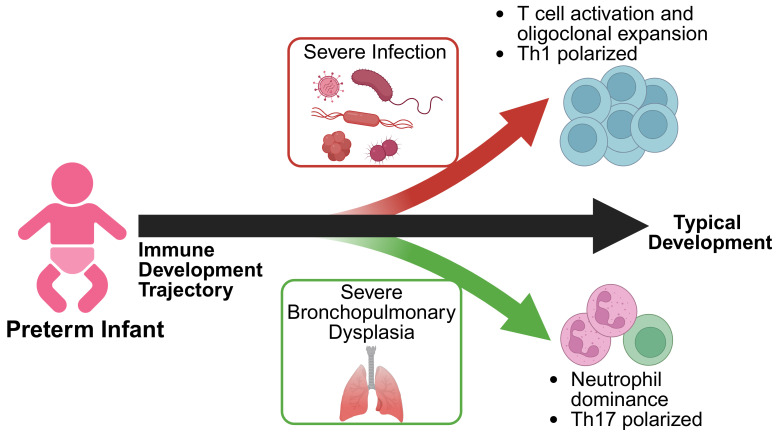

## Introduction

During gestation, the human immune system develops along a programmed trajectory that prepares the fetus for birth (1). This early-life immune system is not simply an immature version of the adult counterpart but rather a dynamic and unique network suited to support organ development and establish homeostasis (2–5). Early-life immune cells arise from distinct hematopoietic precursors (6, 7) and have unique responses to proinflammatory stimuli (8, 9) when compared with adult immune cells. Early-life T cells have a more limited T cell receptor (TCR) repertoire (10–12), are slower to generate long-lived memory populations (13, 14), release different cytokines (15), and polarize in unique patterns compared with adult T cells (16, 17). After term birth, this specialized immune system interfaces with numerous evolutionarily expected stimuli, such as commensal microbes, which ultimately guide the development of the immune system along a stereotypic path toward adult maturity (18, 19).

Preterm birth disrupts this process of immune development, exposing infants to both natural and iatrogenic stressors before immune and organ systems are prepared for extrauterine life (20). These stressors, often required for survival in the neonatal intensive care unit (NICU), include but are not limited to hyperoxia, positive pressure mechanical ventilation, commensal and pathogenic microbes, broad-spectrum antibiotics, and parenteral nutrition (21–25). Ultimately, these exposures can predispose to developmental diseases around the body such as bronchopulmonary dysplasia (BPD) in the lungs, necrotizing enterocolitis in the gut, or systemic bacterial and viral infections, conditions that themselves often require additional supportive yet inflammatory interventions (26). How the rapidly evolving preterm immune system responds to such stressors is poorly defined and often extrapolated from work on term infants, yet there is evidence that such stressors may alter the developmental trajectory of the early-life immune system. Microbial dysbiosis, which occurs more commonly in infants with clinical illness, may change otherwise stereotypic immune development (18, 19, 27, 28). Infants who suffer from clinical illness in early life have an altered risk of allergic, autoimmune, and infectious diseases later in life (28–33). Despite these observations, how preterm birth and its complications durably alter the developmental trajectory of the immune system remains unclear.

Here, we aimed to define the developmental trajectory of the preterm immune system with a focus on interrogating how different comorbidities of prematurity might alter this trajectory. We leveraged residual whole blood derived from complete blood counts (CBC) sent from infants in the NICU and performed serial immune profiling of cells and proteins on 38 preterm infants every 2 weeks, as well as 10 term infants at birth. This granular approach identified immune features that changed over time with gestation, revealing patterns of immune components, cell activation and dynamics, and immune network changes that reflect gestational trajectory and others that diverge due to preterm postnatal life. Moreover, these analyses identified profound immune perturbations associated with preterm comorbidities, such as infection and severe BPD, and highlighted distinct and long-lasting developmental immune changes. These findings illuminate the interplay between immune ontogeny and disease in preterm infants, highlight potential directions for new interventions to improve outcomes, and demonstrate the value of frequent high-dimensional immune profiling in capturing novel and diverse features of preterm disease.

## Results

### Residual blood from preterm infant clinical samples yields high-fidelity, high-dimensional immune information.

**Figure 1:**
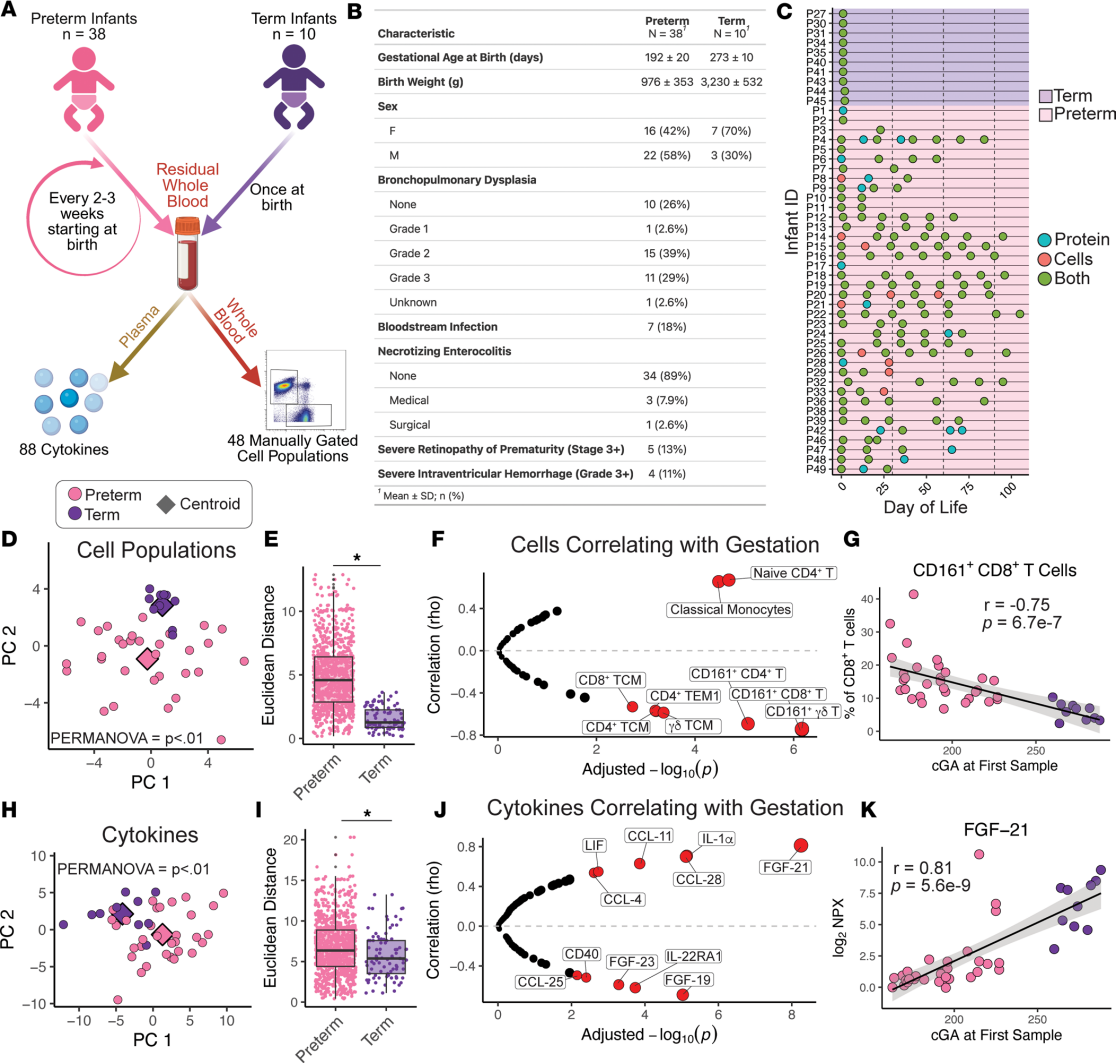
Immune cells and proteins change over gestation. (**A**) Overview of the study design. (**B**) Overview of the clinical features of the infants in the study, including clinical comorbidities during the observation period. (**C**) Each sample collected from each infant in the study. Each dot represents a single sample, and the color of the dot represents whether the sample was used for Olink only (Protein), CyTOF only (Cells), or both. (**D**) PCA plot of cell populations from the first samples of life for each infant. Dots colored categorically by term or preterm. Purple and pink diamonds represent the centroid of term and preterm groups, respectively. (**E**) For cell populations, Euclidean distances between each PCA coordinate within a group to all other points in that group. *P < 0.05 via Wilcoxon rank sum test. (**F**) Plot showing cell populations that significantly correlate with corrected gestational age at birth. Red indicates statistically significant as determined by BH adjusted *P* < 0.01. (**G**) Linear regression of CD161^+^CD8^+^ T cells over gestation. Spearman correlation (rho) and *P* value shown. (**H**) PCA plot of proteins from the first samples of life for each infant. (**I**) Euclidean distances for cytokines between each PCA coordinate within a group to all other points in that group. *P < 0.05 via Wilcoxon rank sum test. (**J**) Plot showing cytokines that correlate with corrected gestational age at birth. Significance determined by BH adjusted *P* < 0.01. Linear regression of FGF-21 over gestational age. Spearman correlation and *P* value shown.

To begin to interrogate preterm immune system development, we first established a pipeline to capture residual whole blood associated with routine clinical assessments of hospitalized preterm infants at the Hospital of the University of Pennsylvania (HUP) Intensive Care Nursery (ICN) (Philadelphia, Pennsylvania, USA), blood that would have otherwise been discarded (Figure 1A). In the HUP ICN, it is common clinical practice to send whole blood for CBC assessment approximately every 2 weeks on all hospitalized preterm infants to monitor for anemia of prematurity. This clinical practice provided an opportunity to test whether residual, unused blood could provide material for detailed assessment of the developing immune system in preterm infants. We enrolled 38 preterm infants born between 23 weeks and 0 days and 32 weeks and 6 days of gestation; we excluded infants with known immunologic or genetic conditions or those who were born to mothers infected with HIV. We also enrolled 10 otherwise healthy term infants who had a single CBC sent for evaluation of hyperbilirubinemia in the newborn nursery at HUP. The infants in the study spanned the gestational age and weight spectrum, and the preterm infants experienced different clinical comorbidities during their time in the HUP ICN (Figure 1B). We then obtained residual blood from the clinical laboratory, with the first sample collected for most infants within 48 hours of life. We continued to collect available blood approximately every 2 weeks, with a minimum of 12 days between samples. We stopped collecting samples once infants reached a corrected gestational age (cGA) of 40 weeks or at discharge if earlier than this age. Infants had different numbers of serial samples collected that were dependent on their total time in the HUP ICN (Figure 1C).

Residual blood samples were processed to obtain plasma for proteomic analysis using the Olink platform and for whole blood mass cytometry by time-of-flight (CyTOF) to interrogate circulating leukocyte populations. Each sample provided ~100–300 μL of remaining material after clinical analysis. CyTOF analysis identified 15 major immune cell populations and 33 subpopulations (Supplemental Figure 1; supplemental material available online with this article; https://doi.org/10.1172/jci.insight.201342DS1). Using plasma from these samples, we were able to resolve 88 immune proteins using Olink. Some collected samples did not have sufficient blood volume for any analysis, and some only had sufficient volume for CyTOF or Olink (Figure 1C).

Residual blood samples were obtained from the clinical lab within 24–36 hours of collection. Given the potential variability in such residual samples, including possible effects of time since collection on leukocyte populations, we first examined the frequencies and activation patterns of major immune cell types, including sensitive populations like neutrophils and eosinophils. These data indicate that, at least within the collection times of this study, the frequencies of major peripheral blood leukocyte populations were stable and did not display major changes or variability associated with time since collection or volume of residual blood available for analysis (Supplemental Figure 2, A and B). Thus, we were able to map longitudinal trajectories of 30-plus immune cell populations for each preterm infant.

### Immune features at birth vary with gestational age.

How the immune cell and protein composition changes throughout gestation has been extensively studied in placental umbilical cord blood (34–36). Although these data provide some insight into the state of the infant immune system during birth, there is emerging evidence that umbilical cord blood does not reliably reflect the immune composition of infant blood immediately after birth, likely because umbilical cord blood is capturing many changes that occur during the birthing process (18, 37). As a result, there are limited assessments of how gestational programming shapes the composition of infant peripheral blood at birth. To begin to investigate the effect of gestational programming on the infant immune system at birth, we first analyzed samples collected within 0–48 hours of birth, typically the first CBC of life, one from each preterm or term infant. To assess the global landscape of the immune cell composition at birth for each infant, we applied principal component analysis (PCA) for circulating leukocyte populations. The overall immune landscape map of term and preterm infants differed substantially, generating divergent PCA centroids (Figure 1D). The interindividual distance between the cell populations of preterm infants was greater than term infants (Figure 1E), potentially reflecting the broader gestational age and weight spectrum of the preterm infant population compared with the term infants or more variable indications for delivery.

We next asked what immune features might contribute the most to this intraindividual immune heterogeneity in early life. We first examined how the frequency of individual immune populations correlated with gestational age. CD8^+^, CD4^+^, and γδ T cells that express CD161 (KLRB1), as well as nonnaive T cell subsets, steadily decreased in abundance from the earliest to latest gestational ages, whereas the proportion of classical monocytes as well as naive CD4^+^ T cells as a fraction of total CD4^+^ T cells increased over development (Figure 1, F and G). CD161 is a marker found on IL-17–producing CD4^+^, CD8^+^, and γδ T cells, and the abundance of CD161^+^ T cells inversely correlates with gestational age in studies of umbilical cord blood (34, 38–40). The identified pattern of these cells here suggests that this correlation continues in the peripheral blood of preterm infants immediately after birth.

To complement the cellular data, we also examined differences in circulating immune proteins in plasma associated with preterm birth and gestational age. Indeed, PCA of plasma cytokines also distinguished term and preterm infants (Figure 1H). Again, the interindividual distance in PCA space based on plasma proteins was greater for preterm infants compared with term infants (Figure 1I). Several cytokines and circulating mediators differed across gestational age (Figure 1J), including fibroblast growth factor (FGF) 19 and 21, which help regulate systemic metabolism (41, 42), increased and decreased, respectively (Figure 1K). FGF-23, a regulator of bone phosphate metabolism (43), decreased. There were also changes in chemokines (CCL4, -11, -25, and -28) and proinflammatory cytokines like IL-1–alpha (IL-1α). These data demonstrate that residual infant blood can effectively capture high-dimensional immune cell and protein composition and highlight how gestational programming continues to imprint major features of the immune system immediately after birth.

### Preterm immune features deviate from gestational development in postnatal life.

**Figure 2:**
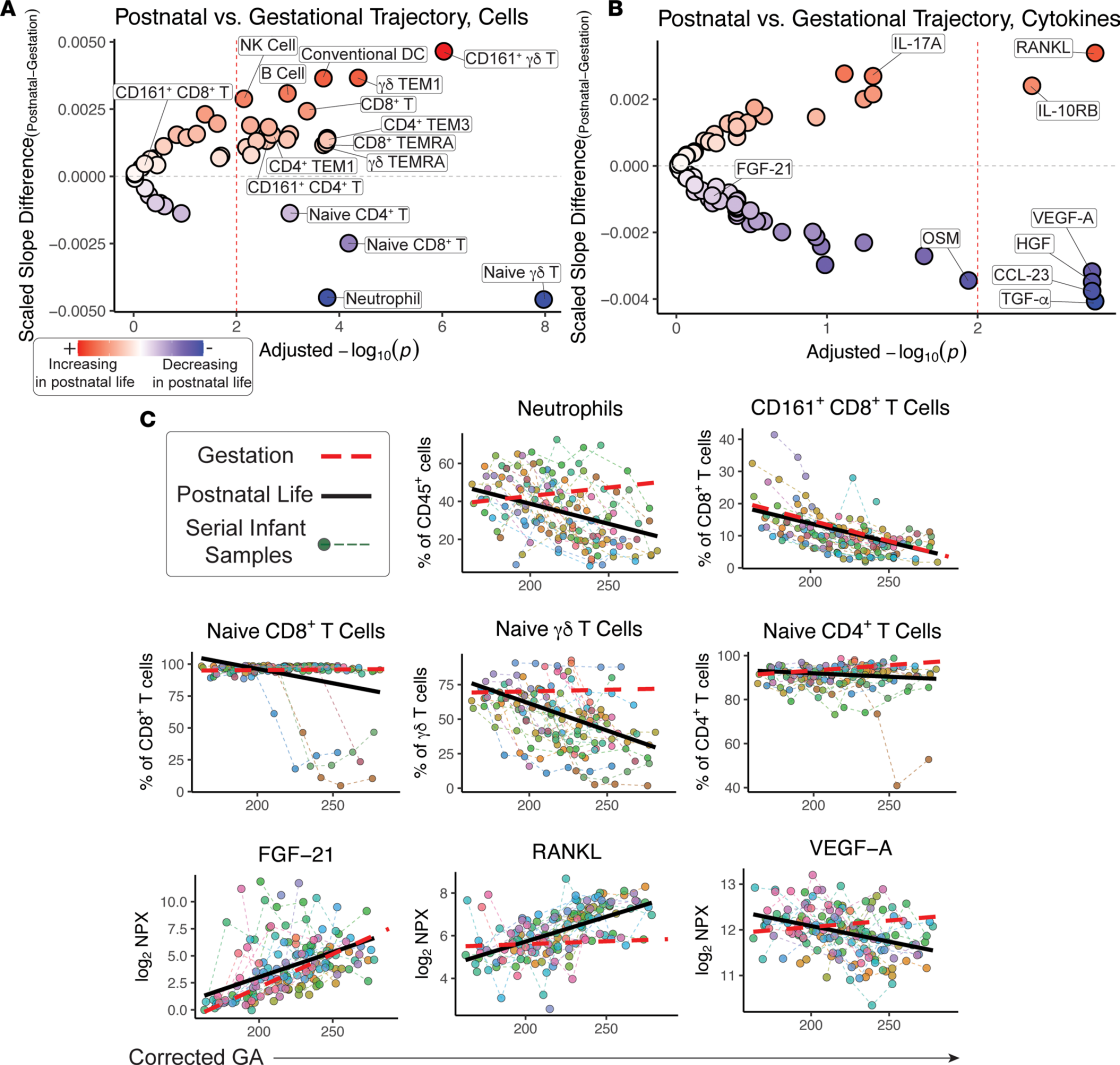
Postnatal life alters the gestational immune trajectory in preterm infants. Linear mixed models were generated for each cell and cytokine. (**A** and **B**) The difference of the scaled slopes from the regression generated from all preterm samples and the regression generated from only at-birth samples, for each cell and for each cytokine. Cell types above the center dashed line represent populations whose slope increases compared with the expected gestational age progression for that cell type at birth, and cell types below the center dotted line represent populations whose slope decreases. Color intensity of the dots (red increased in postnatal life, blue decreased) represents the strength of the slope deviation. Significance set at *P* < 0.01 of BH-adjusted *P* values as determined by the difference in slopes of the 2 linear models. (**C**) Models of specific cell populations neutrophils, CD161^+^CD8^+^ T cells, naive CD8^+^ T cells, naive γδ T cells, and naive CD4^+^ T cells, as well as cytokines FGF-21, RANKL, and VEGF-A, across cGA time. For each plot, the solid black line represents the regression derived from all collected preterm samples. The red dashed line represents the regression derived from the at-birth term and preterm samples only, as calculated in Figure 1. Each dot color corresponds to a single preterm infant, and the small, dashed lines connect serial samples collected from the same infant over time.

Although many aspects of solid organ development continue along a programmed developmental trajectory after preterm birth, conditions of postnatal life can redirect this course. These deviations can manifest as clinical disease and can have long-term clinical consequences (26, 44). However, in contrast to solid organs, relatively little is known about how postnatal life affects the immune system in preterm infants, despite the real possibility that such deviations could also contribute to disease (2, 20, 45, 46). We therefore next asked which features of the immune system diverge from their gestational programming in response to postnatal life in the NICU. To address this question, we compared 2 regression models, one capturing effects of gestational age at birth on immune features and a second based on early neonatal immune development in the NICU. The first model, calculated based on data from Figure 1, is derived from the first sample of life in preterm and term infants and represents the effect of gestational programming on individual immune features. The second model is derived from all postnatal samples collected from preterm infants and represents the effect of postnatal life on these same features. To generate this second model, we built a linear mixed-effects model for each immune feature using all longitudinal samples from each preterm infant. This approach accounted for repeated measures within individuals and incorporated this longitudinal information. We then projected the fixed effects of the model, reflecting change over time since birth independent of individual variation, onto a line. Finally, we compared the scaled slopes from this postnatal line to the line generated from the gestational model (Figure 2A). It is possible that changes in immune cells and proteins could follow nonlinear trajectories. Thus, while this approach allows a general assessment of immune deviation during time in the NICU, this linear analysis may have missed more subtle or nonlinear changes to the immune trajectory. Nevertheless, because both trajectories spanned a similar range of cGAs, as preterm infants exited the study at term-equivalent age and the gestational model included term infants, this comparison allowed us to assess whether each immune feature remained on its expected gestational course or deviated due to postnatal influences.

The frequency of some immune cell populations significantly deviated from their gestational trajectory, whereas others did not. For example, the proportion of neutrophils, naive CD8^+^, CD4^+^, and γδ T cells steadily decreased over time in most infants (Figure 2A). Conversely, the proportion of many subpopulations of nonnaive γδ T cells and conventional T cells, as well as conventional DCs and B cells, increased over postnatal time in the NICU. Some cells, such as CD161^+^CD8^+^ T cells, continued to closely follow a gestational trajectory in postnatal life. This pattern was also found among the plasma cytokines, particularly among proteins related to organ and vascular development such as an increase in the bone-resorption–promoting receptor activator of NF-κB ligand (RANKL), as well as a decrease in cytokines such as hepatocyte growth factor (HGF) and vascular endothelial growth factor A (VEGF-A) (Figure 2B). Many cytokines, such as FGF-21, appeared resistant to deviation from the gestational trajectory in postnatal life.

Immune features deviated from their gestational trajectories with distinct patterns and dynamics. Some changes were observed consistently across the entire preterm cohort over time, whereas others occurred only in a subset of infants (Figure 2C). For example, the proportion of neutrophils, naive γδ T cells, and circulating concentrations of VEGF-A were altered from gestational programming in most preterm infants (Figure 2C). In contrast, there were other patterns, such as a gradual decrease in the proportion of naive CD4^+^ T cells and a sudden and robust drop in the proportion of naive CD8^+^ T cells, that occurred in a smaller number of preterm infants. These data indicate that postnatal life is associated with major deviations from gestational immune system development, with some changes common to most or all preterm infants and others only in a subset of preterm infants, suggesting that specific early-life postnatal conditions may drive immune responses in some infants.

### Severe BPD is associated with divergent Th17 activity.

**Figure 3:**
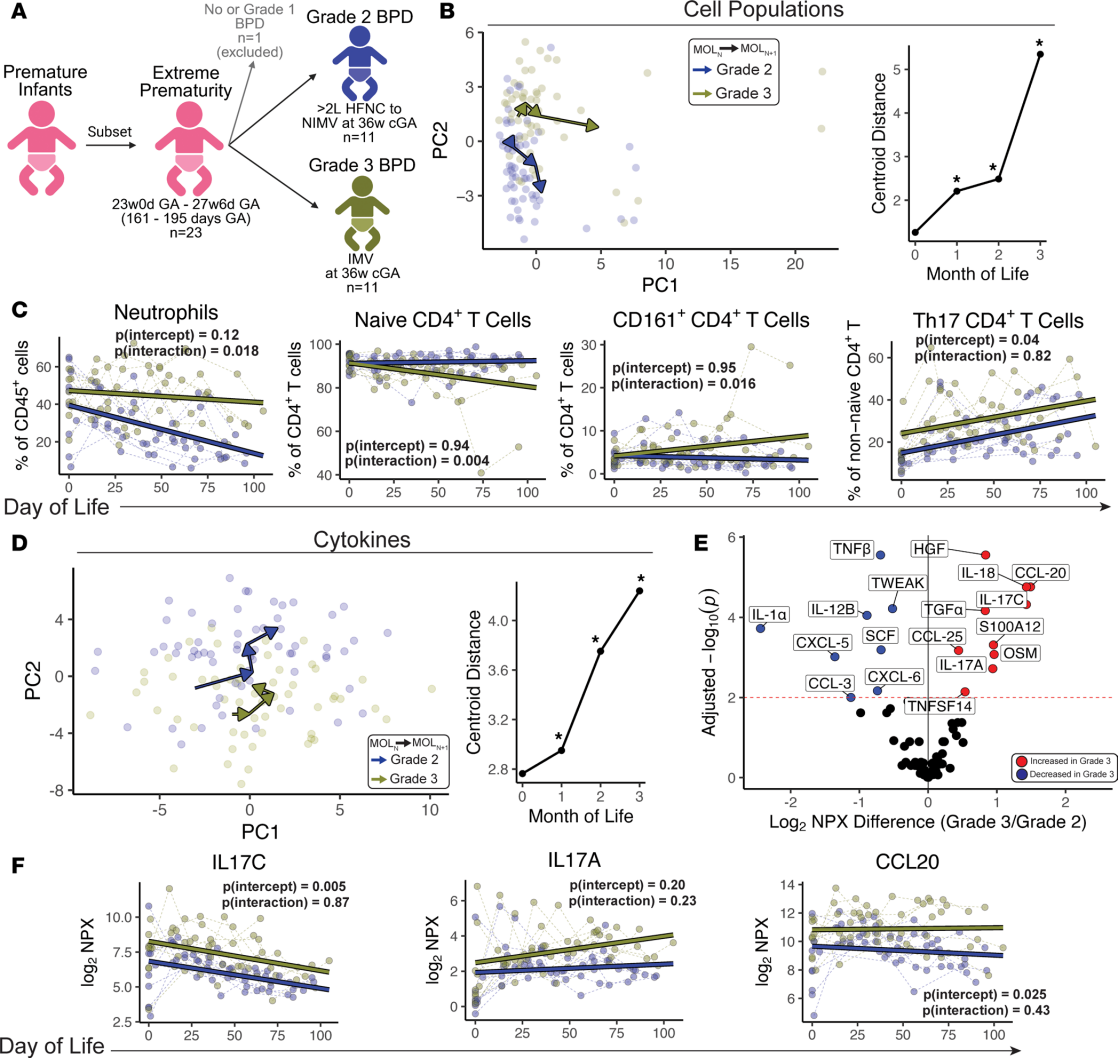
Severe preterm lung disease is associated with a divergent Th17 T cell signature. (**A**) Schematic depicting the infants analyzed for this figure. One infant in the extreme prematurity subset developed Grade 1 BPD and was not included in further analyses in this figure. HFNC, high flow nasal cannula; NIMV, noninvasive mechanical ventilation; IMV, invasive mechanical ventilation. (**B**) PCA of all cell populations in postnatal samples from infants with Grade 2 BPD (blue) and Grade 3 BPD (green). Arrows point to a centroid at each sequential month of life. Euclidean distances between the 2 centroids at each month of life. *P < 0.01 via PERMANOVA. (**C**) Mixed linear regressions of neutrophils, naive CD4^+^ T cells, CD161^+^ CD4^+^ T cells, and Th17 CD4^+^ T cells. Intercept *P* value (representing categorical difference at birth) and interaction *P* value (representing divergence over time) shown for each regression comparing Grade 2 (blue) and Grade 3 (green). (**D**) PCA of all cytokines in postnatal samples from infants with Grade 2 BPD (blue) and Grade 3 BPD (green). Euclidean distances between the centroids at each month of life. *P < 0.01 via PERMANOVA. (**E**) Volcano plot demonstrating cytokines that differ between groups. Cytokines increased in Grade 3 BPD are on the right in red, decreased on the left in blue. (**F**) Mixed linear regressions of IL-17C, IL-17A, and CCL20 with intercept and interaction and *P* values shown.

We next asked whether diagnosed comorbidities of prematurity were associated with some of the deviations of the immune system in postnatal life. The most prevalent preterm comorbidity in the cohort was, expectedly, BPD. BPD is a respiratory disease of prematurity that is defined by distorted lung development and an abundance of proinflammatory cells and proteins in the lung (47, 48). There is evidence that the lung inflammation in BPD reflects a systemic proinflammatory state (49, 50), yet the specific immune perturbations associated with disease remain poorly understood. The incidence of BPD is inversely proportional to gestational age at birth, and most extremely preterm infants develop BPD (51). However, even controlling for gestational age, preterm infants can develop widely different severities of BPD. Infants who develop severe BPD (Grade 3) have very different clinical courses than those with moderate BPD (Grade 2), and the former requires prolonged invasive mechanical ventilation with disproportionate effects on long-term health (52). Thus, to explore how severe BPD is associated with changes in immune development, we focused on a gestational age–controlled subset of preterm infants born at less than 28 weeks gestational age and defined severe or moderate BPD by the level of respiratory support required by each infant at 36 weeks cGA (52) (Figure 3A). The overall immune landscape of preterm infants who developed severe BPD was progressively more different each month after birth from those with moderate BPD in PCA space (Figure 3B). This increasing immune divergence was driven by differences in neutrophils, CD4^+^ T cell subpopulations, and some changes in NK cells and plasmacytoid DCs (Supplemental Figure 3A). To further interrogate how immune cells changed over time between these 2 BPD groups, we constructed a mixed linear model for each cell population. Neutrophil frequencies remained steadily elevated in severe BPD, whereas the frequency of these cells progressively decreased in moderate BPD, contributing to the overall divergence seen each month (Figure 3C). Dexamethasone is frequently used in infants with BPD and can increase the circulating neutrophil pool via neutrophil demargination. Dexamethasone is typically given to infants in short courses to minimize side effects, and 9 samples in our study were collected during active dexamethasone exposure. The samples collected while these infants were receiving dexamethasone had no significant difference in neutrophil frequencies compared with samples from infants who were not receiving the therapy (Supplemental Figure 3B), suggesting that the observed divergence in neutrophil proportion was not due to active dexamethasone administration.

Infants with severe BPD also had a progressive decline in the frequency of naive CD4^+^ T cells, suggesting greater CD4^+^ T cell activation over time (Figure 3C). There was also a progressive increase in the frequency of CD161^+^CD4^+^ T cells (Figure 3C). CD161^+^CD4^+^ T cells are notable, as they are primed to secrete Th17-related cytokines and give rise to nonnaive Th17 T cells (38, 39). In examining the polarization phenotype of all nonnaive CD4^+^ T cells, the nonnaive CD4^+^ T cells of infants with severe BPD reflected a Th17 (CCR4^+^, CCR6^+^, CXCR3^–^) phenotype, but not a Th1 (CCR4^–^, CCR6^–^, CXCR3^+^), Th2 (CCR4^+^, CCR6^–^, CXCR3^–^), or Th1/17 (CCR4^–^, CCR6^+^, CXCR3^+^) phenotype (Figure 3C and Supplemental Figure 3, C and D). It was notable that, whereas neutrophil, naive CD4^+^ T cell, and CD161^+^ CD4^+^ T cell frequencies were similar early and diverged over time, Th17 cells were elevated in severe BPD infants from birth and this difference persisted throughout the time in the NICU.

One of the primary functions of Th17 cells is to promote neutrophil responses at sites of inflammation such as the lung through production of Th17-related cytokines and chemokines (53, 54). The overall pattern of plasma cytokines and inflammatory mediators diverged between infants with severe BPD and infants with moderate BPD (Figure 3D). Among the cytokines increased in severe BPD were IL-17A, IL-17C, and the ligand of the characteristic Th17 chemokine receptor CCR6, CCL20 (Figure 3E). Other proinflammatory Th1-related cytokines, such as IL-12β, lymphotoxin, and IL-1α were lower in infants with severe BPD, highlighting possible polarization toward a Th17 and not a Th1 inflammatory state. Mixed linear model analysis demonstrated that infants with severe BPD had elevated IL-17C and CCL20 at birth and throughout their time in the NICU (Figure 3F). IL-17A did not reach statistical significance in the linear model but trended in the same direction. Together, these data show that the increased neutrophil frequency observed in preterm infants with severe BPD was associated with early-life alterations in Th17-polarized CD4^+^ T cells and Th17-related cytokines, and that these differences may already be present at birth.

### Diverse early-life infections are associated with robust CD8^+^ T cell responses.

**Figure 4:**
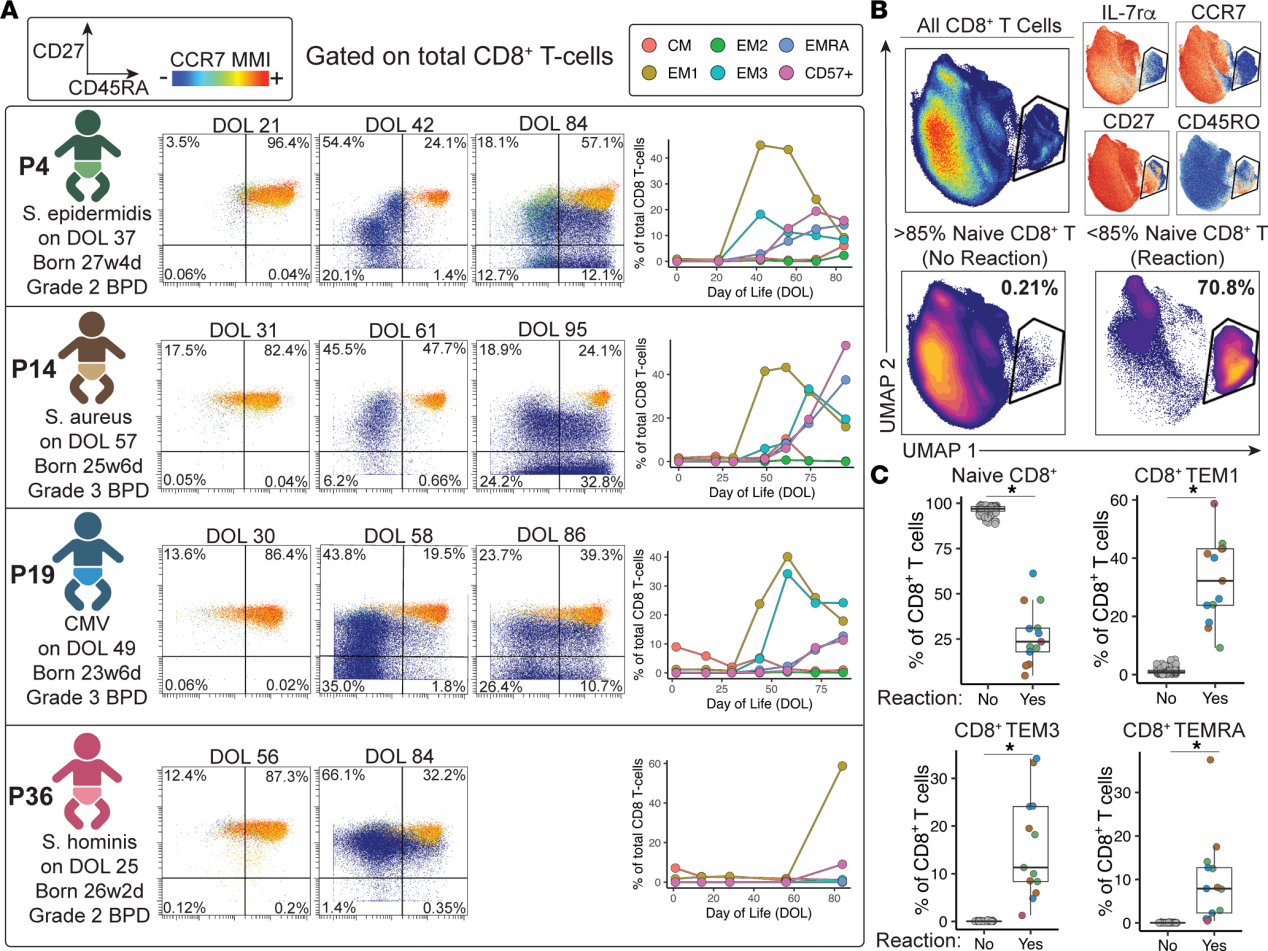
Systemic infection is associated with a sudden, massive, and persistent CD8^+^ T cell reaction. (**A**) Schematic representing the progression of the total CD8^+^ T cell population of 4 infants in the study. Sequential CyTOF plots gated on CD8^+^ T cells shown for each infant. The day of life each sample was collected is denoted above each plot. For each CyTOF plot, *x* axis = CD45RA, *y* axis = CD27, *z* axis (color) = CCR7. Overview plot to the right of each sequence represents specific CD8^+^ T cell subpopulations as they change over time including CM, EM1, EM2, EM3, EMRA, and CD57^+^ cells as a percent of the total CD8^+^ T cell population. (**B**) Density UMAP of all CD8^+^ T cells derived from all samples. Expression of specific markers, IL-7Rα, CCR7, CD27, and CD45RO overlayed on the CD8^+^ T cell UMAP for reference (red indicates high expression, blue indicates low expression). Density UMAP of all samples with > 85% naive CD8^+^ T cells (denoted “No Reaction”) and < 85% naive CD8^+^ T cells (denoted “Reaction”). (**C**) CD8^+^ T cell subpopulations found in samples with and without a reaction. Dot color corresponds to samples from the respective color of the infant as presented in **A**. Samples from infants with no reaction or samples drawn prior to a reaction in gray. *P < 0.05 via BH-adjusted Wilcoxon test.

Although the severity of BPD was associated with some of the postnatal immune perturbations in this cohort, severe BPD alone could not account for all immune changes. Notably, the frequency of naive T cells was similar in infants with severe versus moderate BPD, yet 4 infants — 2 with moderate BPD and 2 with severe BPD — had a sudden and robust decrease in the frequency of naive CD8^+^ T cells during their time in the NICU (Figure 2C). For 3 of the infants, this change in naive CD8^+^ T cell abundance occurred within days of a new onset laboratory-confirmed systemic infection, including blood culture-confirmed *Staphylococcus epidermidis*, blood culture-confirmed methicillin-resistant *Staphylococcus aureus* infection, and blood PCR-confirmed cytomegalovirus (CMV) infection. In 1 infant, P36, this change in CD8^+^ T cells occurred more than a month after both a diagnosis of necrotizing enterocolitis infection and blood culture-confirmed *Staphylococcus hominis* infection. In each of these 4 infants, the typical distribution of 85%–99% CD45RA^+^CD27^+^CCR7^hi^ naive-phenotype CD8^+^ T cells changed abruptly to ~50%–80% of the circulating CD8^+^ T cells displaying markers of activation or an antigen-experienced differentiation state based on CD45RA, CD27, and CCR7 (Figure 4A). This nonnaive CD8^+^ T cell population was composed mostly of effector memory–type (EM-type) cells (CD45RA^–^CD27^+^ or CD45RA^–^CD27^–^). Although individual distributions of CD8^+^ T cell subsets varied, there were some general patterns over time, with effector memory 1 cells (EM1, CD45RA^–^CD27^+^CCR7^–^) expanding first, followed by effector memory 3 cells (EM3, CD45RA^–^CD27^–^CCR7^–^), and then, often weeks later, the emergence of terminally differentiated effector memory reexpressing CD45RA cells (TEMRA, CD45RA^+^CD27^–^CCR7^–^) and CD57^+^ CD8^+^ T cells (Figure 4A). The changes in CD8^+^ T cell subset distribution persisted at least until each infant exited the study, over a month after the associated infection had resolved.

To investigate these neonatal CD8^+^ T cell responses in more detail, we defined any samples from infants in whom the fraction of CD8^+^ T cells fell below 85% naive as having had a reaction. Once an infant first developed a reaction, all subsequent samples also fell below 85% naive CD8^+^ T cells. In samples from these infants, over 70% of the CD8^+^ T cell population shifted to CCR7^lo^, CD27^lo^, CD45RO^hi^, and IL-7Rα^lo^ (Figure 4B). In some patients at some time points, less than 10% of the CD8^+^ T cell population remained naive. CD8^+^ T cell EM3 and TEMRA populations that became the predominant populations in these infants were nearly undetectable in infants that did not have a reaction (Figure 4C).

The microbes associated with these reactions were, in some cases, unexpected. While 1 infant (P19) experienced an early-life infection with CMV, perhaps consistent with the induction of a CD8^+^ T cell response, 2 other infants (P4 and P14) experienced bacterial infections (*S*. *epidermidis* and *S*. *aureus*), 2 infections not typically associated with robust CD8^+^ T cell responses. A fourth infant (P36) had no proximal infection detected at the time of the CD8^+^ T cell response but had experienced a *S*. *hominis* infection at an earlier time point. Although it is possible that infections with clinically undetected intracellular pathogens also occurred concurrently with the confirmed bacterial infections, these events suggest a role for CD8^+^ T cells during both early-life intracellular and extracellular infections in preterm infants. There were no infants without a bloodstream infection who developed a CD8^+^ T cell reaction, and 4 of 7 infants in this study with a confirmed bloodstream infection developed a CD8^+^ T cell reaction. In sum, these observations suggest that robust and dynamic T cell changes may indicate infections even before, or in the absence of, pathogen detection by routine clinical infectious disease testing.

### CD8^+^ T cell reactions to early-life infections are associated with CD4^+^ T cell, γδ T cell, and plasma inflammatory cytokine changes.

**Figure 5:**
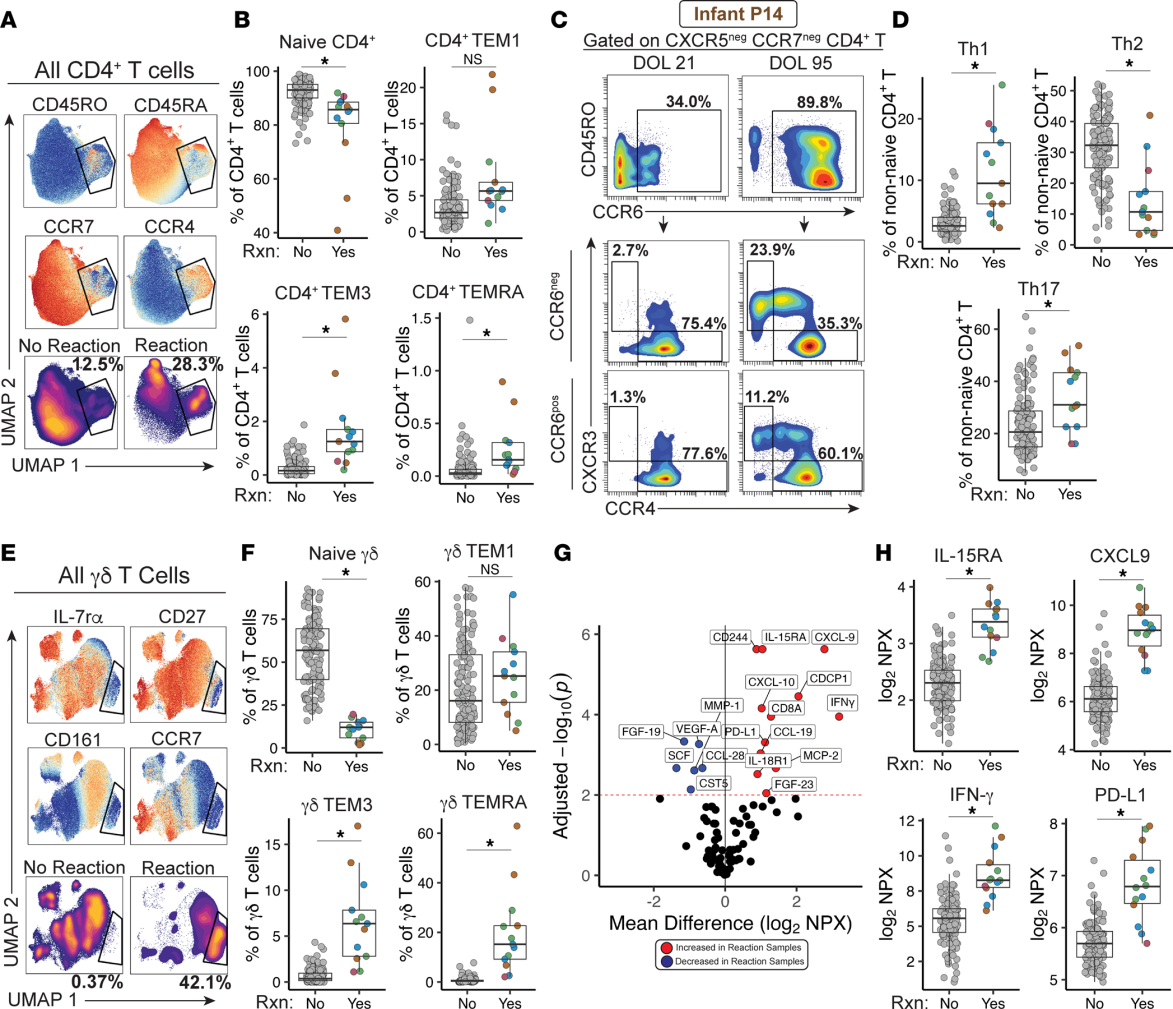
CD4^+^ T cells, *γδ T* cells, and plasma cytokines shift along with the infection-associated CD8^+^ T cell reaction. (**A**) UMAPs of CD4^+^ T cells from all samples showing the expression of different surface markers CD45RO, CD45RA, CCR7, and CCR4 (red indicates high expression, blue indicates low expression), and then density UMAPs representing samples identified as having a CD8^+^ T cell reaction or not. Percentage of cells falling in UMAP gate noted. (**B**) Different CD4^+^ T cell subpopulations (EM1, EM2, EM3, and EMRA) found in samples with and without a CD8^+^ T cell reaction. Dot color corresponds to samples from the respective color of the infant as presented in Figure 4A. Samples from infants with no reaction or samples drawn prior to a reaction in gray. (**C**) Representative CyTOF plots showing the distribution of Th subpopulations of nonnaive CXCR5^–^, CCR7^–^ T cells from 1 sample before a reaction and 1 sample after a reaction from infant P14. (**D**) Average Th1, Th2, and Th17 phenotypes found among samples with and without a reaction. (**E**) UMAPs of γδ T cells from all samples showing the expression of different surface markers IL-7Rα, CD27, CD161, and CCR7 (red indicates high expression, blue indicates low expression), and then density UMAP identified as having a CD8^+^ T cell reaction or not. Percentage of cells falling in UMAP gate noted. (**F**) Different γδ T cell subpopulations found in samples with and without a reaction. (**G**) Volcano plot showing the difference in plasma cytokines from all samples with and without a reaction. Cytokines increased in reaction samples on right. (**H**) Specific plasma cytokines IL-15Rα, CXCL9, IFN-γ, and PD-L1 shown. *P < 0.05 via BH-adjusted Wilcoxon test.

We next investigated whether there were other immune changes in infants with an infection-associated CD8^+^ T cell reaction. Indeed, there was induction of CD45RO^hi^, CCR4^hi^, CD45RA^lo^, and CCR7^lo^ CD4^+^ T cell responses and an increase in EM3 and TEMRA CD4^+^ T cell populations in infants with a CD8^+^ T cell reaction (Figure 5, A and B). CD8^+^ T cell reaction samples also had a reduced frequency of naive CD4^+^ T cells and increased frequency of EM3 and TEMRA subsets (Figure 5B). Although EM1 cells made up the largest population of nonnaive CD4^+^ T cells, these cells were not increased in reaction samples, likely reflecting ongoing EM1 cell elevation over time in nonreaction samples such as those from infants with severe BPD. However, reaction samples had a sharp increase in CCR6^–^CCR4^–^CXCR3^+^ Th1 cells and CCR6^+^CCR4^+^CXCR3^–^ Th17 cells, and a strong decrease in CCR4^+^CCR6^–^CXCR3^–^ Th2 cells (Figure 5, C and D). In addition to this CD4^+^ T cell response, there was also a γδ T cell response in the infants with infection-associated CD8^+^ T cell reactions with a shift to CD27^lo^, CCR7^lo^, CD161^lo^, and IL-7Rα^lo^ γδ T cells (Figure 5E). Using analogous marker subsets for αβ cells to look at γδ T cell phenotype, there was little change in γδ T EM1-like cells, but the frequency of EM3-like and TEMRA-like γδ T cell populations were increased (Figure 5F). Thus, whereas γδ T cell subpopulations change in most infants after birth with a gradual increase in CD161^+^ and nonnaive γδ T cells, only infants with an infection-associated CD8^+^ T cell reaction have γδ T cell responses resulting in differentiation into subpopulations analogous to αβ T EM3 and TEMRA populations.

These T cell responses in infants with infections were also associated with many changes in plasma cytokines and inflammatory proteins, including an increase in many proteins related to CD8^+^ T cell and CD4^+^ Th1 responses, such as IFN-γ, IL-15Rα, CXCL9, CXCL10 (CXCL9 and CXCL10 are ligands for the Th1 receptor CXCR3), soluble IL-18R1, and soluble PD-L1, together with lower FGF-19, VEGF-A and others (Figure 5, G and H). Thus, in a subset of preterm infants in this study, systemic viral or bacterial infection was associated with robust CD8^+^, CD4^+^, and γδ T cell responses and was associated with circulating inflammatory mediators, leading to durable changes in the circulating adaptive immune compartment for at least several weeks after resolution of infection.

### Infection-associated T cell responses are oligoclonal and progressive.

**Figure 6:**
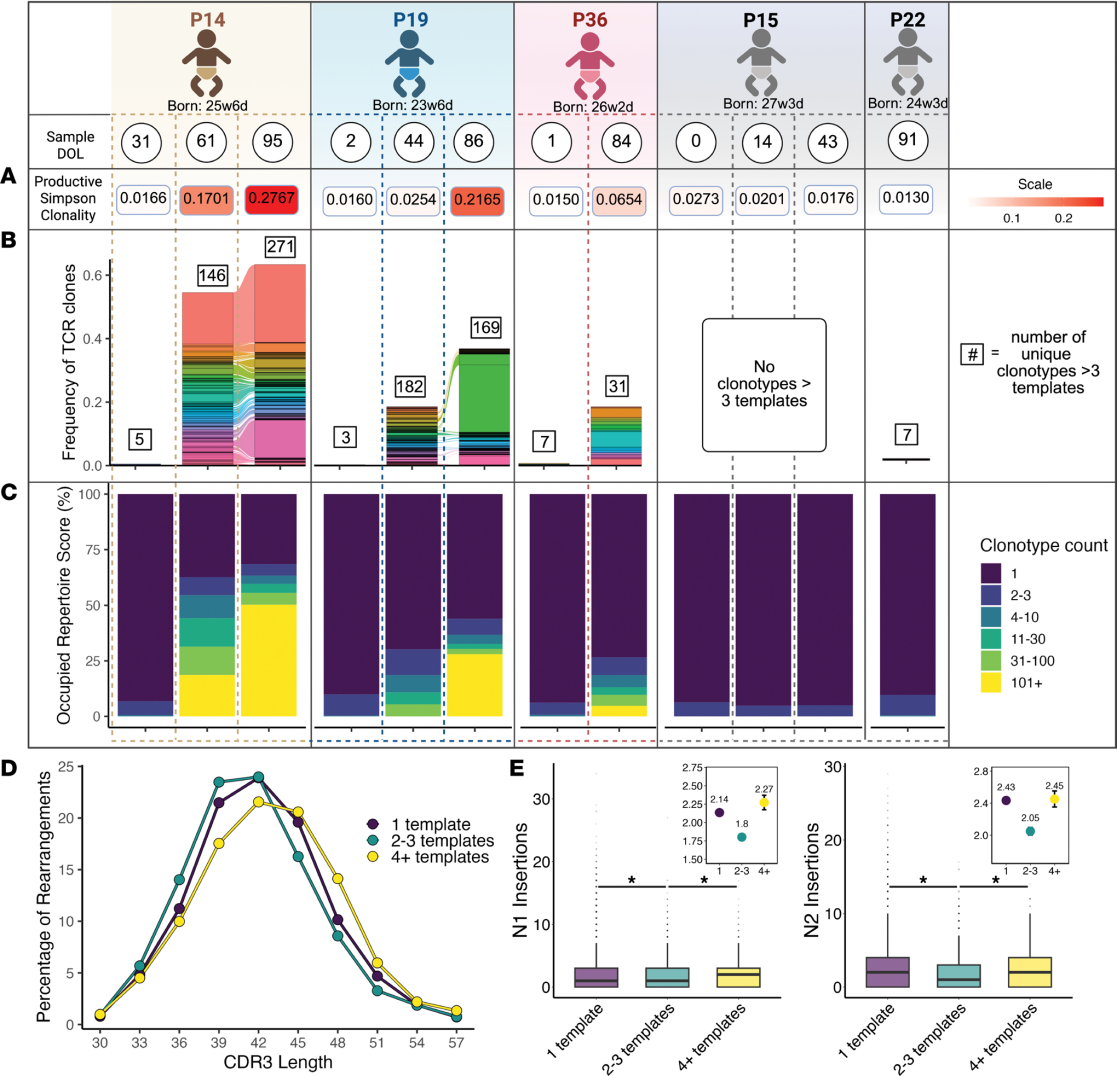
Massive first-in-life T cell reaction is oligoclonal. TCR-seq analysis of select samples from infants with a T cell reaction. Information from each sample can be seen as a subcolumn under the infant from which it was derived. (**A**) The productive Simpson clonality index of each sample. (**B**) The frequency of specific TCRs that were found to have greater than 3 clonotypes within the sample. Each color represents a unique TCR sequence. Alluvia connecting the colors between samples represent the same TCR sequence that was found in both samples. (**C**) The occupied repertoire score, which is the percent of the sample that is dominated by TCRs of a given clonotype count. (**D**) The distribution of CDR3 lengths of TCRs within each clonotype count bin. All samples are grouped. (**E**) N1 and N2 insertions among each unique TCR within each clonotype count bin. Zoomed in means for each group in graph inset. All samples are grouped. BH-adjusted *P < 0.05 via Kruskal-Wallis test with post hoc Dunn’s test.

Given such a robust shift in T cell differentiation state in the blood of certain infants with infection, we next investigated whether this event represented a nonspecific polyclonal T cell response, as has been reported in neonatal T cells (55, 56), or was more oligoclonal suggesting an antigen-driven response. We isolated DNA for TCR sequencing from time points before and after an infection and T cell response from 3 of the infants with a reaction. The blood samples from infant P4 were not available for this analysis. We also performed TCR sequencing for 2 other age- and BPD-matched infants who did not develop a bloodstream infection. TCR clonality increased progressively in each infant after infection (Figure 6A), and the TCR pool became clonally dominated soon after the infection with increasing clonality over the following month (Figure 6, B and C). Prior to infection, or in infants with no infection, there was limited to no TCR expansion. In infants with infection, although some expanded clones were not captured at every time point, there were detectable expanded clones that were shared between serial time points (Figure 6B). Queries of public TCR databases did not identify consensus putative antigen specificities for these expanded TCRs (data not shown). Thus, the T cell responses observed in preterm infants with infection were associated with robust TCR oligoclonal expansion.

We next asked whether the highly expanded TCR clones varied in their complementarity-determining region 3 (CDR3) length or number of N insertions compared with unexpanded or mildly expanded clones. In general, preterm CDR3 lengths are shorter, with fewer N insertions, compared with mature adult TCRs (10). In infants with infection-associated expanded TCR clones, the CDR3 lengths of expanded clones tended to be longer than those of less expanded TCR clones (Figure 6D). The number of N-insertions in TCR clones with 4 or greater copies was also increased compared with clones with 2 or 3 copies, though TCRs represented by only a single TCR copy had similar N-insertions to the highly expanded clones (Figure 6E). TCR clones with 2 or 3 copies can be found with similar frequency in all the samples sent for TCR analysis, whereas TCR clones with greater than 4 copies were uniquely found in samples with infection reaction. Thus, despite a restricted early-life TCR repertoire (10, 57), preterm infant infection with CMV or extracellular bacteria in the NICU was associated with robust TCR clonal expansion and persistent reshaping of the TCR repertoire.

## Discussion

Recent work has begun to dissect the unique dynamics of the preterm human infant immune system (18, 28, 58–60). A major gap in our understanding of early-life human immune development, however, is how postnatal stressors and clinical disease perturb preterm immune development. To address this question, we developed a high-dimensional longitudinal immune profiling approach to interrogate the trajectory of the preterm immune system during postnatal life in the NICU. Using this approach, we captured early-life immune development changes in preterm infants highlighting rapid changes in neutrophils, T cells, and inflammatory mediators when infants were forced to adapt to birth prior to full gestation. In addition, we captured the effect of 2 common clinical comorbidities of prematurity on immune development. First, infants who developed severe BPD were characterized by a progressively Th17-dominated immunologic signature. Second, some infants who developed viral or extracellular bacterial infection had a robust oligoclonal T cell response dominated by CD8^+^ T cells, along with CD4^+^ T cell responses, γδ T cell responses, and type 1 cytokine changes. Many of these durable immune changes were continuing to evolve when the observational period of the study ended and, thus, may have immune imprinting effects that extend beyond this period of clinical observation, though longer-term analysis of immune change was not possible in the current cohort, given the method of obtaining samples. If there are durable imprints of these early life immune events, it will be interesting to explore possible mechanisms including epigenetic imprinting, changes to the TCR (or BCR) clonal hierarchy, and/or persistence of inflammatory signals.

Th17 CD4^+^ cells have a central role in many autoimmune and inflammatory disorders (61) and may also contribute to pathophysiologic changes in preterm lungs (62). Neonatal CD4^+^ T cells can readily undergo Th17 polarization in a manner that is inversely related to gestational age (63). Our observations that the proportion of CD161^+^CD4^+^ T cells inversely correlates with gestational age in infant blood, a finding also seen in studies of cord blood (34, 39), are consistent with findings that preterm neonates can readily undergo Th17 polarization. It is worth noting that the risk of severe BPD also inversely correlates with gestational age (51); thus, extremely preterm infants predisposed to Th17 polarization have a developmental lung state most at risk of severe BPD. The data here further show that infants who ultimately develop severe BPD have progressive increases in Th17 CD4^+^ T cells, circulating Th17 cytokines, and neutrophils over time, all characteristic of a Th17 signature. It was not possible to perform T cell functionality assays given the limited material available. As a result, the source of Th17-related cytokines in circulation cannot be definitively ascribed to the Th17-like T cells observed, and other cell types such as IL-17C–producing epithelial cells (64) could contribute. Nevertheless, it is tempting to speculate that extremely preterm infants who activate CD4^+^ T cells early in life have a bias toward inducing and then reinforcing the Th17 program (perhaps with contributions from other cell types), thereby increasing the likelihood of neutrophilic inflammation, which could damage an already immature lung architecture. For example, in mouse models of neonatal BPD-like lung inflammation, blockade of the Th17 system reduces the degree of inflammation and damage in the lung (65, 66). Additionally, histologic chorioamnionitis at birth, a risk factor for developing severe BPD, is associated with an increase in Th17 cells in the cord blood, implicating type 17 responses in preterm inflammation (67, 68). Current methods of reducing inflammation in BPD, such as dexamethasone, have numerous side effects that make them difficult to use as an ongoing therapy in infants (69). The IL-17 axis offers a more specific potential target for intervention in BPD, and there exist multiple, safe, approved therapies that target the Th17 system (70). Whether blockade of the Th17 axis is a viable antiinflammatory approach to reduce the risk or severity of BPD warrants further study.

In addition to severe BPD, systemic infection in a subset of infants in our cohort was associated with unique and profound changes to the T cell pool. In part because our scavenged blood design allowed us to frequently profile preterm infants, we captured multiple robust first-in-life T cell reactions in real time. These findings add to the evidence that the preterm immune system, despite its restricted T cell repertoire and resistance to certain inflammatory stimuli, can be highly reactive and proinflammatory, mounting vigorous adaptive immune responses in some settings of infection (4, 5). Robust CD8^+^ T cell activation in infants has been observed following infection with several viral infections, including acquired CMV (71), congenital CMV (72), and HIV (73). These studies reported that durable alterations in the CD8^+^ T cell compartment could be found even 12 months after infection in term infants (71). These new data in preterm infants, in addition to identifying coordinated CD8^+^ T cell, CD4^+^ T cell, γδ T cell, and plasma protein changes, suggest extensive oligoclonal T cell activation might also be a feature of severe bacterial infection, in addition to viral infection. Indeed, studies in rodents have demonstrated that neonatal CD8^+^ T cells are critical for controlling extracellular bacterial infections (74). Moreover, neonatal T cells have the ability to secrete CXCL8, a neutrophil-attracting chemokine, whereas adult T cells do not (15). These observations suggest that neonatal T cells may play a larger role in conditions where neutrophil activity is needed, such as bacterial infections.

There were no concomitant viral infections diagnosed in the infants with a laboratory-confirmed bacterial blood culture. It remains possible, however, that an undiagnosed concurrent viral infection was present in these settings. It is also notable that many of the infants in our study who reached 2 months of age likely received the full complement of vaccinations around this time. Although we could not coordinate sample acquisition around the timing of these vaccinations, immunologic reactions to vaccination were not obvious in our dataset, suggesting that infection, rather than early-life vaccination, was needed to trigger these robust adaptive immune responses. Whether these T cell responses in preterm infants are unusual compared with responses in term infants remains an open question. Although infant responses to bacterial infection have been examined (75, 76), few studies have examined persistent T cell responses in this settings. Thus, future studies that directly compare T cell responses to bacterial (or viral) infections in term and preterm infants will be revealing. Regardless of the trigger or whether these types of responses are unique to preterm infants, the physiologic relevance of such reactions, including how children who experienced these immune responses might respond to future infections or noninfectious immune challenges, warrants further study. While there was no clear association between this infection-associated reaction and the development of severe BPD in this study, investigations into whether this T cell reaction can influence the development of other comorbid conditions are warranted. Interestingly, the differentiation pattern and T cell subset distributions in these preterm infants are somewhat comparable with those found in the healthy adult CD8^+^ T cell compartment (77), suggesting that this sudden shift to an expanded, differentiated, and TCR matured CD8^+^ T cell pool may be an event that occurs in all humans at some point during life. If true, how and when this event occurs may have meaningful immunologic consequences. Whether these altered T cell responses and TCR repertoires in early life reflect differences in thymic output that will normalize over time also remains unknown. Nevertheless, these findings reinforce the need for long-term studies investigating whether early life immune events are associated with future clinical disease.

There are likely many other immunologic or clinical conditions, not captured in this cohort, that are associated with durable changes in the immune trajectory. Detailed information for each preterm infant — such as the indication and mode of preterm delivery, whether infants were born in the setting of histologic chorioamnionitis, or whether infants were exposed to early antibiotics or supraphysiologic oxygen levels — could not be captured in the current cohort due to limitations on collected clinical data. These additional data, if available in future cohorts, may further inform the analysis of the immune trajectory beyond gestational age alone and allow for a robust multivariate analysis. Even after birth, there are other major comorbidities of prematurity, including necrotizing enterocolitis, severe interventricular hemorrhage, or severe retinopathy of prematurity, which were not captured with enough power to make biologic conclusions here but warrant future investigation given that they may be associated with their own unique immune perturbations (78, 79). Preterm infants are also exposed to numerous stressors in the NICU that we did not track in this study. One exposure that is of particular interest is commensal microbes. Olin et al. reported that infants with microbial dysbiosis have greater interindividual diversity compared with infants with a healthy microbiome, and they suggest that otherwise stereotypic immune development may be altered in these infants (18). Dysbiosis has been associated with a number of immunologic changes early in life that can precede diagnosis of disease later (19, 27, 80). In animal models, rodents with dysbiosis have increased Th17 cell–driven asthma that persists even after normalizing their microbiome (81). When considered with our findings of a progressive Th17 polarization in BPD, a condition in which asthma-like airway hyperreactivity is widely prevalent (82), this set of work suggests that the infant microbiome may play a key role in predisposing infants to disease-specific immunologic skewing. Beyond the microbiome, Tan et al. also recently demonstrated that the type of milk fed to preterm infants, another factor we did not track in this study, can change immune development (60). While the current work provides a foundation for interrogating the preterm infant immune system and dynamic trajectory, there are numerous unmeasured factors that may drive immunologic diversity that warrant further study.

Overall, these data document major immunological changes associated with common clinical events of prematurity and suggest durable consequences to immune development. Moreover, we reveal that different early-life comorbidities can change immune trajectories in unique ways. Thus, while each preterm infant immune system will ultimately become “adult,” the path it takes to get there can be shaped and may be rewired by factors such as infection or severe lung disease. Each of these unique trajectories may reflect ongoing illness, as with evolving severe BPD, or past illness, as with systemic infection. Defining how these early-life immunologic trajectories ultimately influence the development of childhood and even adult immune health will be an important goal.

## Methods

### Sex as a biological variable.

This study enrolled both male and female infants, and similar findings are reported for both sexes.

### Study overview.

Preterm infants admitted to the HUP ICN were considered eligible for the study if they met criteria of born between 23 weeks and 0 days and 32 weeks and 6 days. Infants were excluded from the study if they had any known genetic diagnosis at birth, any severe congenital anomalies, or were born in the setting of HIV. After enrollment, residual unused blood from the first CBC of life, often sent by clinicians in the first 24 hours of life, was obtained from the clinical lab and deidentified. Whole blood was collected from the HUP clinical lab 24–48 hours after CBC analysis was completed. Residual blood collected for these studies would otherwise have been discarded. A minimum of 12 days was required between serial sample collection for each infant. Blood was recurrently obtained based on the availability of samples until an infant was discharged or met 40 weeks cGA. All blood was collected in K2-EDTA vacutainers. Samples were stored at room temperature in the clinical lab until processing.

### Clinical data collection.

Selected clinical data as approved by the IRB were obtained for each infant including age, sex, and weight at birth; time and results of each CBCs sent; the diagnosis of major comorbidities as appearing in the electronic medical record; time and results of blood; urine and cerebrospinal fluid infectious testing sent on each infant; and the start and end dates of dexamethasone or hydrocortisone exposure. All clinical data was stored on a secured REDCap database contained within the University of Pennsylvania system.

### Plasma cytokine analysis.

In total, 50 μL of whole blood was centrifuged at 2,000*g* for 15 minutes, and plasma removed and stored at –80°C until analysis via the Olink target 96-inflammation kit (Olink). Data were reported as normalized protein expression values (NPX). The NPX values for 2 batch runs were normalized using bridging samples present in both analyses. Cytokines were excluded from subsequent analysis if > 25% of the samples analyzed were below the limit of detection for the assay or the cytokine failed quality control metrics. Eight cytokines were ultimately excluded for these technical reasons, resulting in 88 included cytokines.

### CyTOF analysis.

Up to 300 μL of whole blood was stained with the MaxPar Direct Immunophenotyping Assay (MDIPA) lyophilized antibody cocktail (Standard BioTools Inc.) for 30 minutes at room temperature (83). The assay requires 300 mL of whole blood. If the available blood volume was less than 300 μL, heat-inactivated pooled human AB serum was added to meet 300 μL. After 30 minutes, 420 μL of PROT1 proteomic stabilizer buffer (SmartTube Inc.) was added, followed by 10 minutes incubation at room temperature. Samples were then stored at –80°C until batched analysis. Samples were thawed and RBC lysis was performed using Thawlyse buffer (Smart Tube Inc.) as per manufacturer’s recommendation. In total, 125 nM iridium (Cell-ID Intercalator-Ir, Standard BioTools Inc) in MaxPar Fix Perm buffer (Standard BioTools Inc.) was then added and samples were stored at 4°C until further processing for acquisition. A batch control sample, prepared by staining a single large batch blood sample drawn from a healthy adult donor, was included in each batch of sample acquisition. Samples were washed twice with MaxPar Cell Staining Buffer (Standard BioTools Inc), followed by 2 washes with Cell Acquisition Solution+ (CAS+, Standard BioTools Inc), filtered through 35 μm nylon mesh and loaded on CyTOF XT mass cytometer (Standard BioTools Inc.) for data collection. Daily instrument QC and tuning was performed using EQ Six (EQ6) element calibration beads (Standard BioTools Inc). EQ Four (EQ4) element calibration beads (Standard BioTools Inc.) were added at a 10% v/v to the samples that were suspended at a concentration of 1 million per mL in CAS+ buffer. The CyTOF Software v9.1, data acquisition software, used signals from EQ4 bead channels to normalize data from analyte channels. Cells were injected into the detector at ~500 events per second and at a flow rate of 30 μL per minute. Manual gating of immune cell populations was performed using OMIQ software (Dotmatics) to identify immune populations for analysis. All raw CyTOF data were arcsin scaled prior to gating. All full gating scheme is provided in Supplemental Figure 1.

### TCR sequencing.

DNA was isolated from fixed immune cells remaining after CyTOF analysis with the PureLink Genomic DNA Mini Kit (Invitrogen). Briefly, cells were decrosslinked and incubated with proteinase K overnight. The next day samples were incubated with RNase and spun into a DNA collection tube, washed, and eluted. DNA purity and quality was assessed via NanoDrop (Thermo Fisher). Isolated DNA from each sample was sent for TCR sequencing (Adaptive Biotechnologies). Initial data were analyzed using the ImmunoSEQ Analyzer 2.0 toolkit and exported for further analysis and plotting in R.

### Statistics.

All deidentified data were exported from REDCap and stored in secure Microsoft Excel data frames and analyzed via R. Correlations for immune features over gestation were performed using a Spearman’s rank coefficient. All PCA plots generated were scaled. PCA plots were statistically analyzed via 1-way PERMANOVA tests using the *vegan* package in R. All linear mixed models were generated and analyzed using the *lme4* package in R. To construct a fixed-effects regression line as used in Figure 2, a prediction grid was constructed spanning the range of observed cGA days and predicted the fixed effect estimates from the fitted mixed model with the random (infant-specific) effect set to zero. TCR data were plotted using the *ggalluvial* package. All *P* values were Benjamini-Hochberg (BH) corrected as indicated, and thresholds of significance are reported in each figure. All other data were plotted and statistically analyzed using R packages, including *ggplot2*, *dplyr*, *tidyr*, *ggpubr*, *reshape2*, and *stats*. Summary figures were created using BioRender with an academic license.

### Study approval.

This study was reviewed and approved by the University of Pennsylvania IRB. As this study used residual blood from samples collected for clinical purposes and had no patient-facing interactions, this study was approved with a waiver of consent, which limited potential selection bias. Each category of clinical information gathered from the medical record was outlined and reviewed by the IRB prior to collection, and all data were stored in a secure deidentified REDCap database.

### Data availability.

The values underlying the graphed data can be found in the Supporting Data Values document. All raw data and code used for analysis are available from the authors upon request.

## Author contributions

BAF and EJW conceived of and designed the research study. BAF, EJW, and KL wrote and sponsored the IRB protocol. BAF, MLM, S, AP, ZR, ZM, CD, SN, MCK, and CC acquired the data from collected samples. BAF, DM, MLM, ML, ARG, and EJW developed the data analysis approaches. BAF and EJW wrote the manuscript. All authors critically reviewed the manuscript.

## Conflict of interest

EJW is a member of the Parker Institute for Cancer Immunotherapy. EJW is an advisor for Arpelos Bio, Arsenal Biosciences, Coherus, Danger Bio, IpiNovyx, New Limit, Marengo, Pluto Immunotherapeutics, Related Sciences, Santa Ana Bio, and Synthekine. EJW is a founder of Arpelos Bio, Arsenal Biosciences, Danger Bio, and holds stock in Coherus.

## Funding support

This work is the result of NIH funding, in whole or in part, and is subject to the NIH Public Access Policy. Through acceptance of this federal funding, the NIH has been given a right to make the work publicly available in PubMed Central.

- The Thrasher Research Fund Early Career Research Award (to BAF)
- American Academy of Pediatrics Marshall Klaus Award (to BAF)
- NIH grants AI155577, AI115712, AI117950, AI108545, AI082630, AI149680, HL145754 (to EJW)
- Parker Institute for Cancer Immunotherapy (to EJW)
- The Mark Foundation (to EJW)
- Arguild Foundation and philanthropic support to the UPenn Immune Health Innovation fund (to EJW)

## Supplementary Material

Supplemental data

Supporting data values

## Version 1 04/21/2026

In-Press Preview

## Version 2 06/08/2026

Electronic publication
