# Supplementary material for: Perturbation of the preterm human immune system in early life
Source: JCI Insight. 2026 Apr 21;11(11):e201342. doi: 10.1172/jci.insight.201342 (PMC13313540; doi:10.1172/jci.insight.201342)
Supplement: Supplemental data [file jciinsight-11-201342-s231.pdf]

Supplemental Figure 1: Gating Strategy
Cells in bold were analyzed as a proportion of total CD45+ cells. Cells underlined were analyzed as a proportion of the parent cell in bold above it.

> CD45+ Cells (CD45+, DNA+)
>> Granulocytes (CD66b+, CD45 lo-mid)
>>> **Neutrophils** (CD294-, CD16hi)
>>> **Eosinophils** (CD294+, CD16lo)
>> Mononuclear Cells (non-Granulocytes)
>>> Non-monocyte, non-NK cell (CD14-, CD56-)
>>>> Non-T (CD3-, IL3R-)
>>>>> **B cells** (CD19+, CD20+)
>>>>>> Naïve B cell (IgD+, CD27-)
>>>>>> IgD negative Memory B cell (CD27+, IgD-)
>>>>>> IgD positive Memory B cell (CD27+, IgD+)
>>>>> CD20 negative (CD20-)
>>>>>> **Plasmablast (CD38+, CD27+)**
>>> Non-T cell, non-B cell (CD3-, CD19-)
>>>> Non-NK cell (CD56-, CD20-)
>>>>> **Monocytes (CD11c+)**
>>>>>> Classical Monocytes (CD14+, CD16-)
>>>>>> Intermediate Monocytes (CD14+, CD16+)
>>>>>> Non-Classical Monocytes (CD14-, CD16+)
>>>>> **Basophils (IL-3R+, HLA-DR-)**
>>>> Non-Monocyte (CD14-, CD20-)
>>>>> Dendritic Cells (HLA-DR+, CD56-)
>>>>>> **Plasmacytoid Dendritic Cells (CD11c-, IL-3R+)**
>>>>>> **Classic Dendritic Cells (CD11c+, IL3R-)**
>>>>> **NK Cells (CD56+, HLA-DR-)**
>>>>>> CD56 bright NK Cells (CD56 hi, CD16-)
>>>>>> CD56 dim NK cells (CD56 lo-mid)
>>>>>>> Early NK Cells (CD57-)
>>>>>>> Late NK Cells (CD57+)
>>> T cells (CD3+, CD19-)
>>>> **NK T cells (CD56+)**
>>>> **γδ T cells (TCR γδ+)**
>>>> ab T -cells (TCR γδ-)
>>>>> **CD8 T cells (CD8+, CD4-)**
>>>>> **CD4 T cells (CD8-, CD4+)**
>>>>> **Double positive T cells (CD8+, CD4+)**
>>>>> **Double negative T cells (CD8-, CD4-)**
--The following applies to CD8+ T cells, CD4+ T cells, and γδ T cells each--
>>>>>> CD161+ T cells (CD161+)
>>>>>> CD57+ T cells (CD57+)
>>>>>> CD27+ T cells (CD27+)
>>>>>>> Effector Memory 1 T cells (CCR7-, CD45RA-)
>>>>>>> CCR7+ T cells (CCR7+)
>>>>>>>> Central Memory T cells (CD45RO+, CD45RA-)
>>>>>>>> Naïve T cells (Non-Central Memory T cells)
>>>>>> CD27- CD45RA- T cells (CD27-, CD45RA-)
>>>>>>> Effector Memory 2 T cells (CCR7+)
>>>>>>> Effector Memory 3 T cells (CCR7-)
>>>>>> T-effector memory re-expressing CD45RA cells (CD27-, CD45RA+)

Supplemental Figure 1: Gating Strategy (cont.)
Representative gating strategy from a single infant P14 sample.

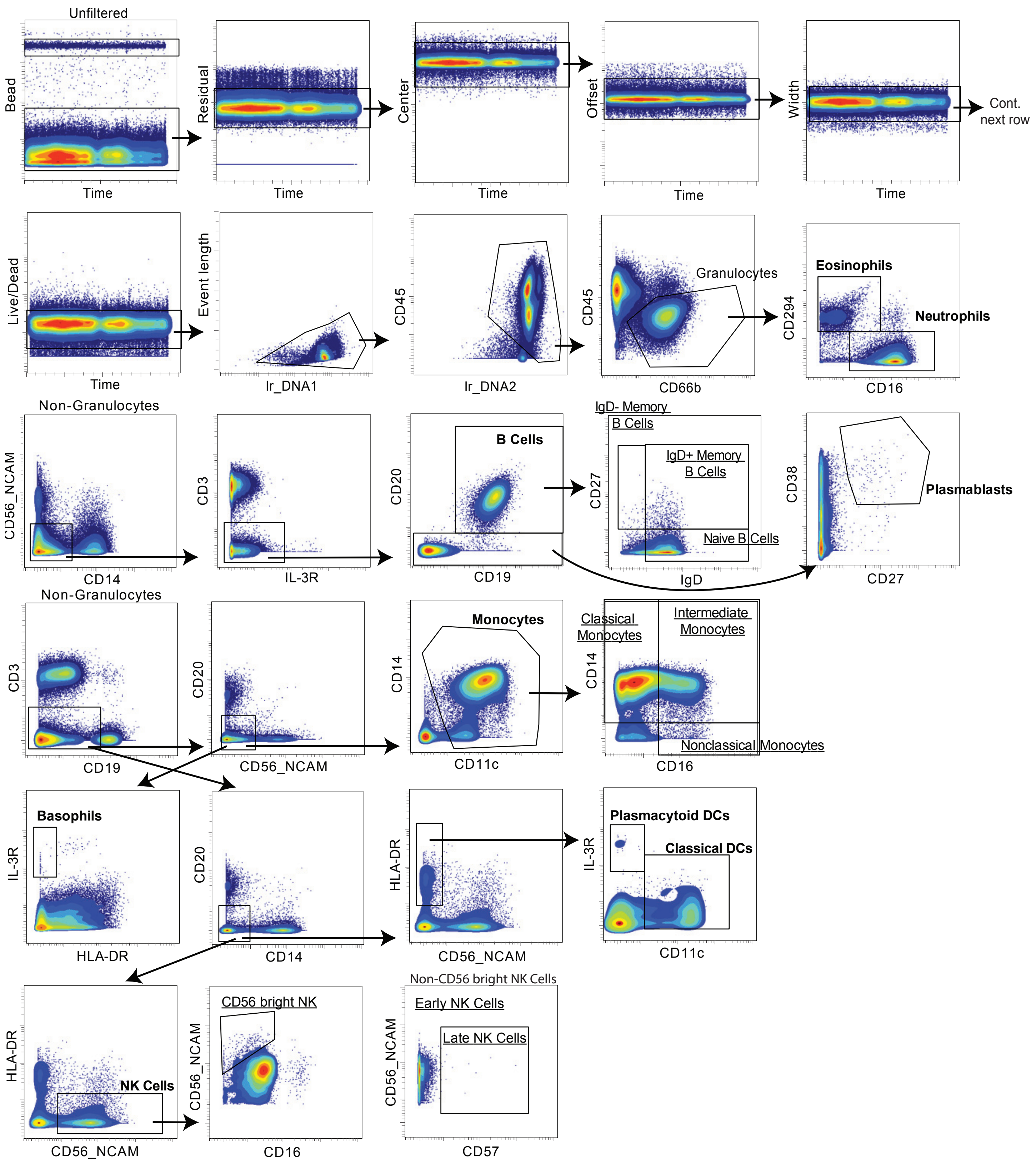

Continued on next page

Supplemental Figure 1: Gating Strategy (cont.)

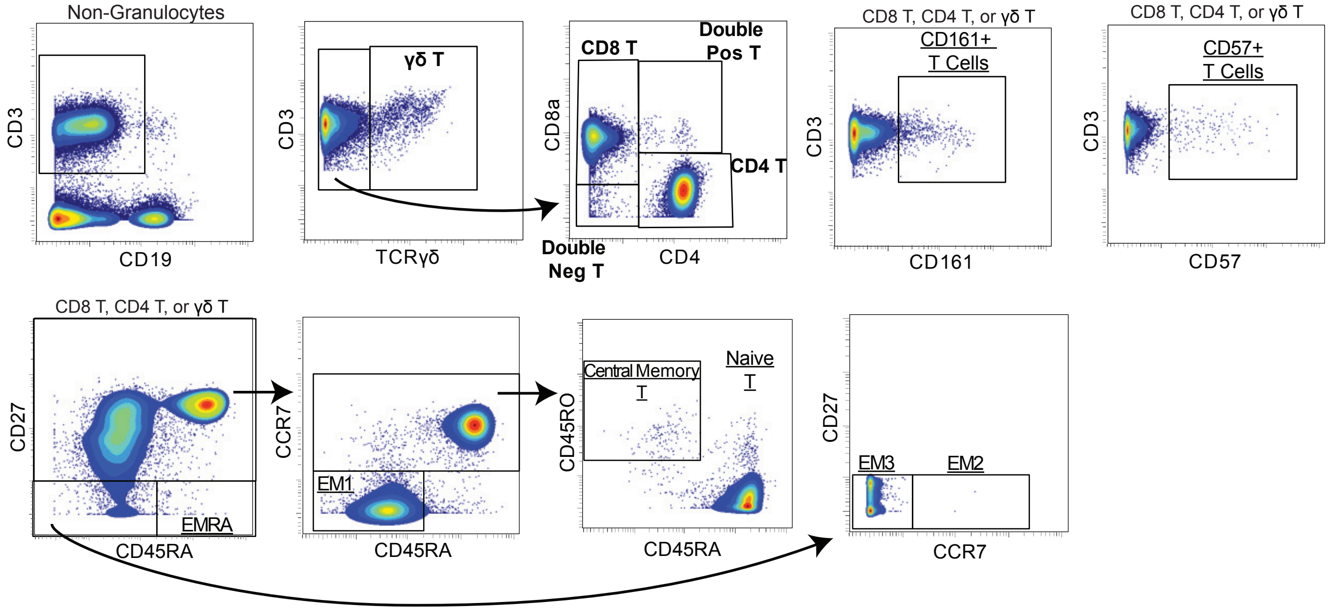

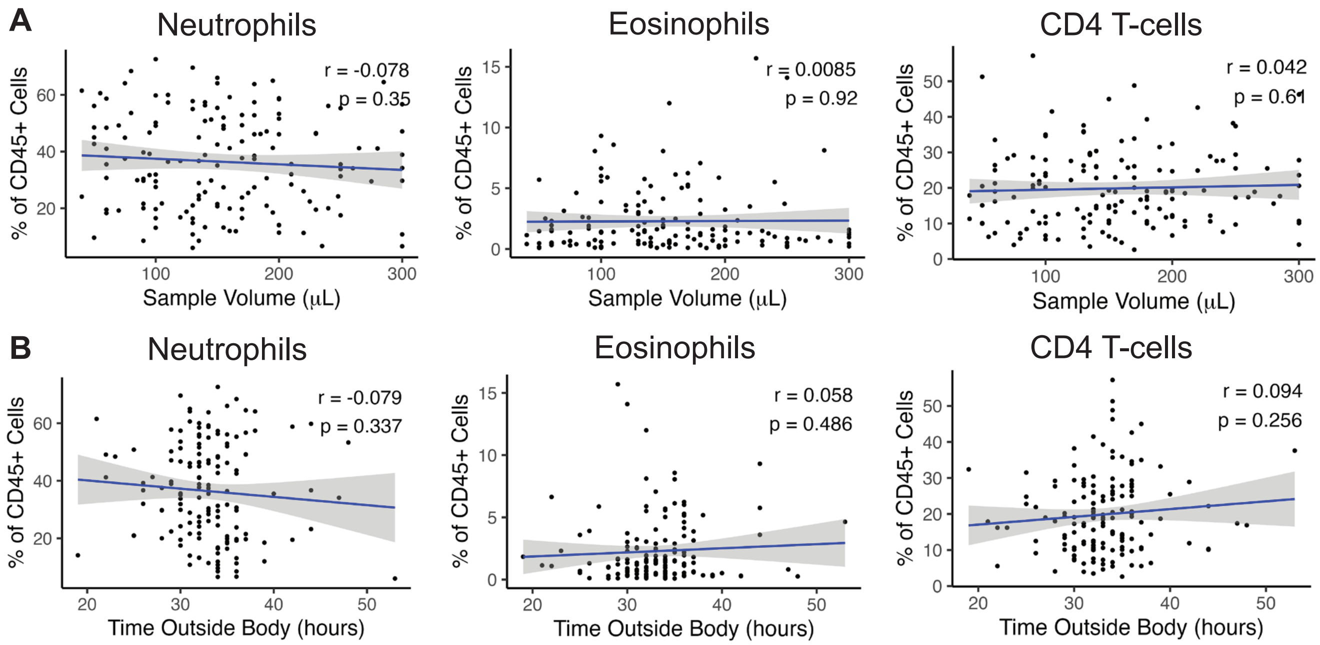

<u>Supplemental Figure 2: Sample volume and time outside of body does not impact overall cell composition</u>

A) Neutrophils, eosinophils, and $CD4^+$ T cells proportions in all samples over a range of residual sample volumes.
B) Cells in all samples over the time each sample rested in the clinical lab before processing for this study.

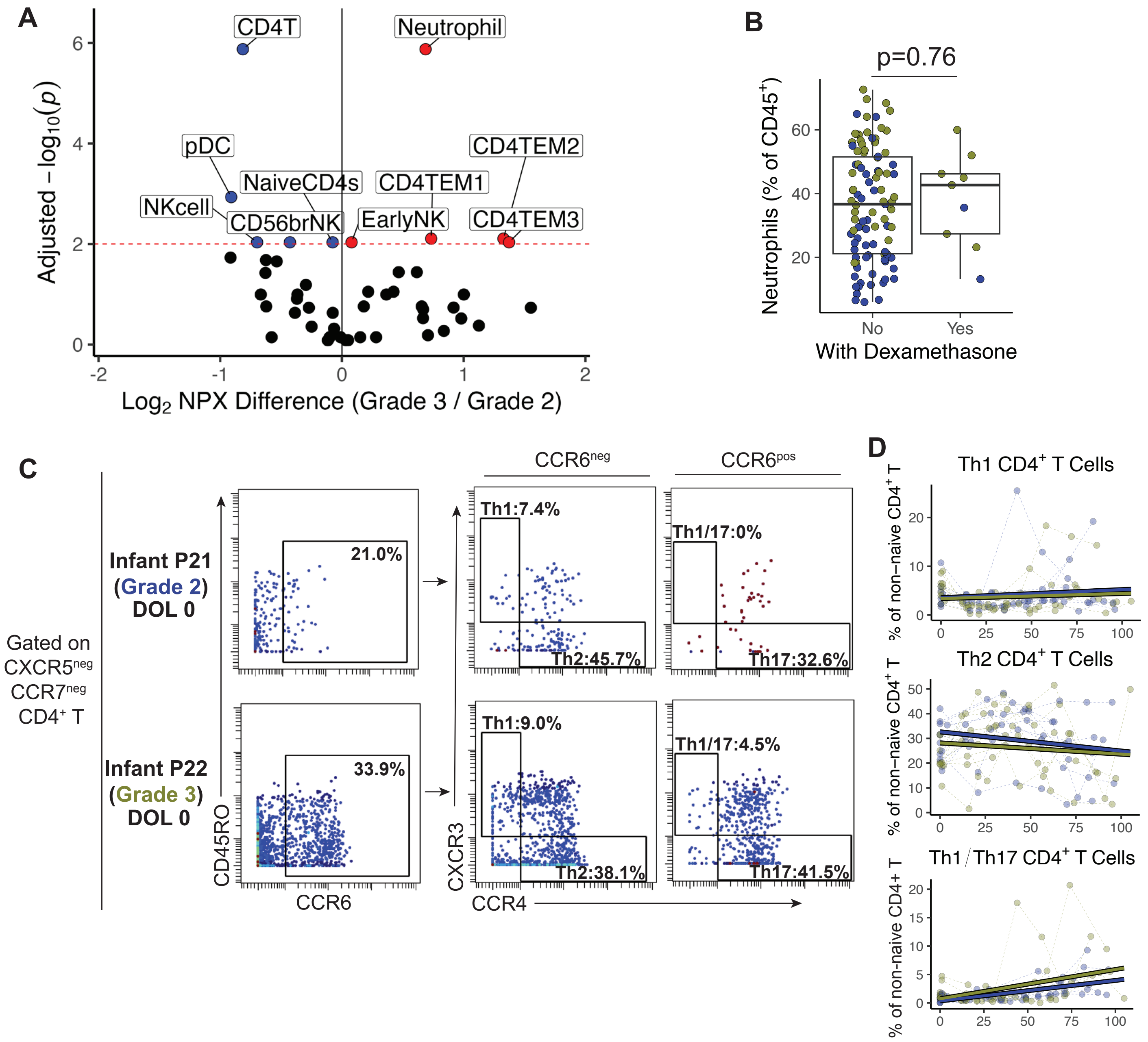

Supplemental Figure 3: Infants with severe BPD have altered neutrophil and $CD4^+$ T cell profiles

A) Volcano plot demonstrating cell populations that differ between infants with Grade 3 and Grade 2 BPD. B) Proportional neutrophil levels in each sample by whether the sample was collected during active dexamethasone administration. Infants with severe (green) and moderate (blue) BPD shown. P-value derived from Wilcoxon test. C) Representative CyTOF plots showing the distribution of Th subpopulations within non-naïve $CXCR5^-$, $CCR7^-$ T cells from two infants, one ultimately developing moderate BPD (P21) and one severe BPD (P22), at one timepoint on DOL 0. D) Mixed linear model of Th1, Th2, and Th1/Th17 cells as a proportion of non-naïve $CD4^+$ T cells over time from severe BPD (green) and moderate BPD (blue).
